# Vulnerabilities of the Open Platform Communication Unified Architecture Protocol in Industrial Internet of Things Operation

**DOI:** 10.3390/s22176575

**Published:** 2022-08-31

**Authors:** Dong-Hyuk Shin, Ga-Yeong Kim, Ieck-Chae Euom

**Affiliations:** 1 System Security Research Center, Chonnam National University, Gwangju 61186, Korea; 2 Department of Data Science, Chonnam National University, Gwangju 61186, Korea

**Keywords:** open platform communication (OPC) unified architecture (UA), vulnerability discovery framework, vulnerability analysis, industrial control system, industrial Internet of Things

## Abstract

Recently, as new threats from attackers are discovered, the damage and scale of these threats are increasing. Vulnerabilities should be identified early, and countermeasures should be implemented to solve this problem. However, there are limitations to applying the vulnerability discovery framework used in practice. Existing frameworks have limitations in terms of the analysis target. If the analysis target is abstract, it cannot be easily applied to the framework. Therefore, this study proposes a framework for vulnerability discovery and countermeasures that can be applied to any analysis target. The proposed framework includes a structural analysis to discover vulnerabilities from a scenario composition, including analysis targets. In addition, a proof of concept is conducted to derive and verify threats that can actually occur through threat modeling. In this study, the open platform communication integrated architecture used in the industrial control system and industrial Internet of Things environment was selected as an analysis target. We find 30 major threats and four vulnerabilities based on the proposed framework. As a result, the validity of malicious client attacks using certificates and DoS attack scenarios using flooding were validated, and we create countermeasures for these vulnerabilities.

## 1. Introduction

### 1.1. Background and Motivation

As processes become more precise and complex, the importance of communication to transmit data between the equipment controlling the process increases. Before the advent of open platform communication (OPC), industrial communication protocols, such as Modbus or PROFINET, were different for each equipment manufacturer. Hence, all equipment required for the process was unified with products of the same manufacturer using the same communication protocol. However, because communication between devices was essential according to the demand for various functions, the interface capability has been increased through intensive investment in collecting communication protocols through partnership with equipment manufacturers.

In addition, a human–machine interface (HMI) with an integrated protocol was created, requiring the user to purchase and use the HMI program to reduce the communication and convenience burden of process management. However, although the communication problem was solved in this manner, the user was forced to only use products from major manufacturers linked to the HMI or had to purchase an expensive license for communication even in an environment that did not require an HMI. In addition, the need for a standard communication protocol with high versatility, rather than the HMI, was emphasized for small-scale or late-stage equipment manufacturers because of communication entry barriers created by major manufacturers and HMI companies [1].

A technology born in response to this demand, OPC, was introduced in 1996. It is an industrial standard protocol created to facilitate communication between all equipment controlling a process. Based on component object model/distributed component object model (COM/DCOM), a technology developed by Microsoft for Windows, OPC enables data communication between production systems and automation devices from other vendors via a Windows PC. In addition, the OPC Unified Architecture (UA), an integrated architecture, was released by the OPC Foundation in 2008 to improve the initial OPC, which can only be operated based on the Windows operating system. As a result, the OPC UA with improved interoperability can be deployed on Windows, Linux, VxWorks, and various real-time operating systems. Hence, it is evaluated as a machine-to-machine protocol for industrial automation with the advantage of being unaffected by the operating system.

### 1.2. Problem Setup

The OPC UA protocol was designed with security in mind according to the OPC UA security architecture specified in OPC 10000-2 [2]. Furthermore, the Practical Security Recommendations were presented by the OPC Foundation [3]. The German Federal Office for Information Security (BSI) published the Security Analysis Report for the OPC UA [4]. However, to use the OPC UA, the user must actively configure the security settings, and the system might be vulnerable or unsecured due to improper security settings. Therefore, 13 OPC UA vulnerabilities reported from July 2017 to May 2021 were investigated. This investigation included cases of exploiting vulnerabilities in OPC UA applications. Recently, cases of exploiting the vulnerabilities of the NET Framework in OPC UA applications have been increasing. Therefore, although the OPC UA protocol design is secure, it can be significantly affected by various security setting options, resulting in actual security threats [5].

### 1.3. Goal and Purpose

In this study, operationally possible control system vulnerabilities were analyzed through virtual simulations using the OPC UA protocol. After developing a five-step analysis framework, the environment configuration, data flow analysis, threat modeling, and attack verification were performed according to the framework Then, a response plan was mapped to each attack scenario.

A previous study evaluated the security level of Internet-connected OPC UA deployments and configurations and found that 92% of OPC UA servers were misconfigured because of missing access controls, disabled security features, use of dedicated cryptographic primitives, or reuse of certificates. Although the author mentioned that the configuration of the OPC UA is complicated [6], other studies have suggested that such issues are attributed to the OPC UA standard supporting insecure operations [7].

Moreover, studies have demonstrated denial-of-service (DoS) attacks, packet sniffing, and man-in-the-middle (MiTM) attacks through attack simulations on OPC UA networks [8,9,10,11,12,13,14]. However, they focused on the impact assessment of industrial Internet of Things (IoT) systems and verified possible attack types for known security threats specified in the OPC UA system specifications. The current study identified the data flow for the environment configuration based on a real industry model using the OPC UA protocol. Then, the STRIDE (spoofing, tampering, repudiation, information disclosure, Dos, and elevation of privilege) threat modeling technique was applied to possible threats during the operation to identify them and construct and demonstrate attack scenarios.

The contributions can be summarized as follows:

- We propose a framework for discovering vulnerabilities and creating countermeasures for analysis targets.
- We selected the OPC UA as an analysis target and applied it to the framework through a case study to derive results.
- We analyzed the attack scenario from the attacker’s viewpoint to identify the attack path and create countermeasures.

This paper details the cybersecurity attack scenarios that can occur during operations by implementing a virtual plant using the OPC UA, an industrial control protocol. The sections are organized as follows. Section 1 introduces the necessity of OPC according to the need for integrating control protocols used in industrial control systems (ICSs), and explains the problem and motivation for this study. Section 2 analyzes the security elements of major control protocols based on an understanding of ICSs. In addition, Section 2 explains and discusses the background of the OPC protocol, the OPC UA, the communication structure between a client and server, and the security architecture. Section 3 proposes a framework for vulnerability discovery and countermeasures. The framework consists of five steps: environment configuration, structural analysis, threat modeling, vulnerability analysis, and countermeasures. Section 4 presents a case study conducted to apply to the proposed framework. The analysis target is the OPC UA, including its environment. We derived vulnerabilities through analysis and verified them through attack scenarios. Finally, Section 5 summarizes the study and discusses future research.

## 2. Related Work

### 2.1. Trends in Protocols for Industrial Control

**Figure 1:**
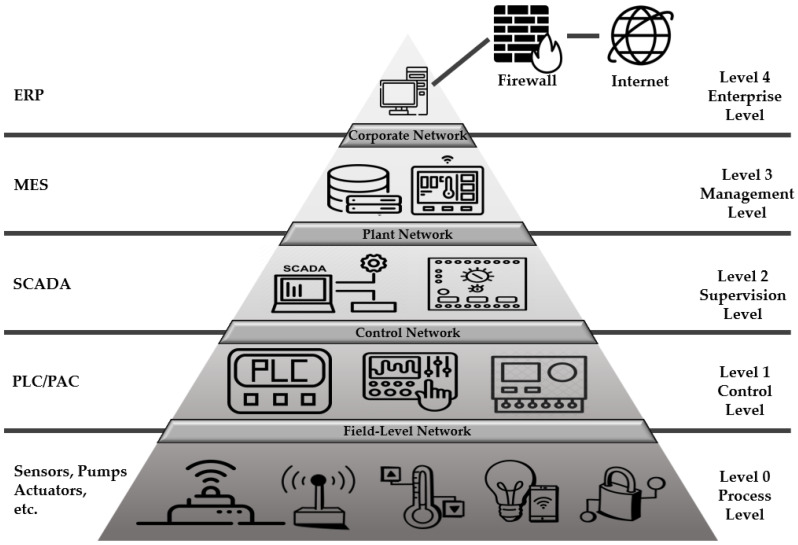
Structure of an ICS.

Potential security threats to ICSs increase as digital transformation and connectivity through information technology (IT) technologies increase. In addition, as the interest in smart factories has recently increased, many articles and materials on ICS security can be found. In fact, ICS security operations have been in progress for a long time, but the interest in security is on the rise after the risks and ripple effects of many recent breaches have been recognized. The ICS refers to several control systems, including supervisory control and data acquisition (SCADA), distributed control systems (DCSs), programmable logic controllers (PLCs), and field devices used in industrial production. Figure 1 illustrates the structure of the ICS specified in IEC 62264 [15], an international standard for integration.

Then, ICS security standards are addressed in a broad context. Considering that the production technology varies from system to system, the ICS configuration varies for each manufacturing plant. Table 1 summarizes the comparison results of the main characteristics and security goals of the ICS with those of the IT system.

Unlike the general IT environment, an ICS is an environment in which safety and continuity are more important than security. In the case of a security incident in an ICS, the physical operation can be controlled, so safety and continuity cannot be guaranteed. Therefore, physical damage may occur, which may cause personal injury. Cyberattacks on ICSs cannot simply be regarded as cyber damage and can cause human casualties during a production process interruption or accident. However, the security of ICSs has not yet been strengthened, and many vulnerabilities exist. Table 2 and Table 3 summarize the results of identifying the characteristics of the major protocols used in the control system and possible network security threats and analyzes the security requirements reflecting the characteristics of the control system.

**Figure 2:**
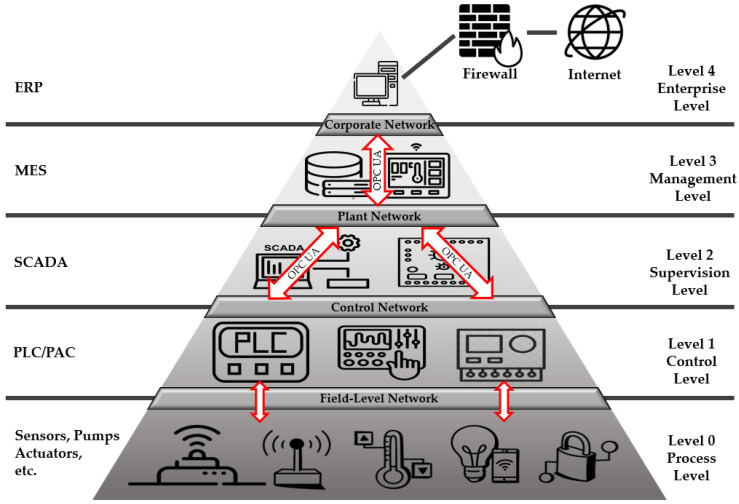
Architecture with integrated OPC in facility automation.

According to Hardware Meets Software (HMS) Networks of industrial control protocol trends in 2021, the industrial Ethernet (e.g., PROFINET and Modbus-TCP) accounts for approximately 65% of the network [16]. The OPC UA, a standard for integrating these industrial Ethernet communication protocols, provides vertical and horizontal communication from field-level sensors and actuators to enterprise-level enterprise resource planning (ERP), as depicted in Figure 2.

### 2.2. OPC Protocol

The OPC is an international industry standard communication protocol created to facilitate communication between all equipment controlling a process. The OPC’s object linking and embedding (OLE) function can use each object of the window in various ways in the application program. From the description of OLE, OPC Classic, the original OPC, works based on Windows. Subsequently, the OPC UA was developed, bridging the gap between the Internet protocol (IP)-based world of IT and the production site. It supports various operating systems (OSs), not limited to Windows, enables communication between various platforms, and supports a wide range of platforms from embedded systems or devices to mobile, enterprise systems, and the cloud. In terms of security, OPC UA offers (1) user and application instance (software) authentication, (2) confidentiality and integrity through message signing and encryption, and (3) availability through minimal processing prior to authentication. In addition, it is (4) defined for OPC UA operation, providing auditability via audit events, and is different from OPC Classic. 

The OPC technology consists of a server and client. The server provides an interface for obtaining measurement values of network devices and turning individual motor switches on and off. A client is a user program that reads, writes, and controls data provided by the server. The server connects to the equipment and operates in the background, and the client primarily comprises the HMI and transmits the device status and user control input to the server.

#### 2.2.1. OPC Classic

Moreover, OPC Classic communicates using Microsoft’s DCOM technology, which communicates between components on network computers and is the underlying protocol of OPC. Although the first COM technologies were only available for homogeneous systems, DCOM enables interprocess interactions on heterogeneous systems. In addition, OPC Classic was primarily used by HMI and SCADA systems to access data from devices from multiple vendors and other types of automation hardware using one defined software interface provided by the hardware vendor. With the successful application of OPC Classic in numerous products, it has been used as a standardized interface between automation systems at different levels. However, if one wants to use a standard, such as OPC, more products become unavailable because of the OPC’s COM dependency or remote access using DCOM [17].

#### 2.2.2. OPC UA

The OPC UA emerged owing to the barriers of Microsoft’s technologies COM and DCOM, which are problems for OPC Classic. In addition, because data access between DCOM and TCP is often strictly restricted by various security policies, security issues have been raised. Consequently, the OPC UA has emerged to substantially replace all existing COM-based specifications without losing functionality or performance. In addition, complex systems must meet the constraints of having scalable modeling capabilities and all the requirements for platform-independent system interfaces. Moreover, it can be extended from embedded systems in SCADA and DCS to manufacturing execution system MES and ERP systems [18]. The main requirements are as follows:

1. Operation on Windows PC and various automation devices;
2. Exchange of structured data, semantic information, simple numerical values, and memory data;
3. Enhanced security.

**Figure 3:**
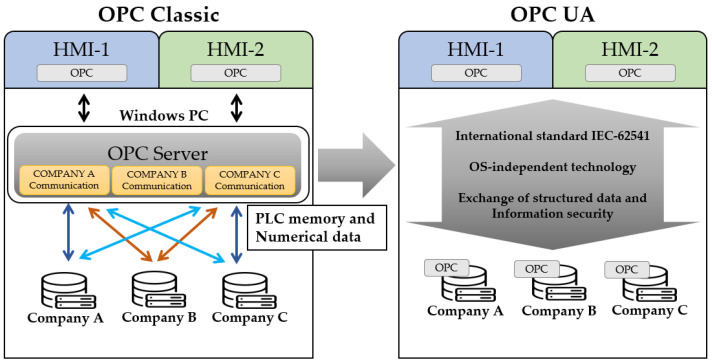
Comparison between OPC Classic and OPC UA.

The differences in the communication methods of OPC Classic and OPC UA are compared in Figure 3 [19].

#### 2.2.3. OPC UA Security Model

**Figure 4:**
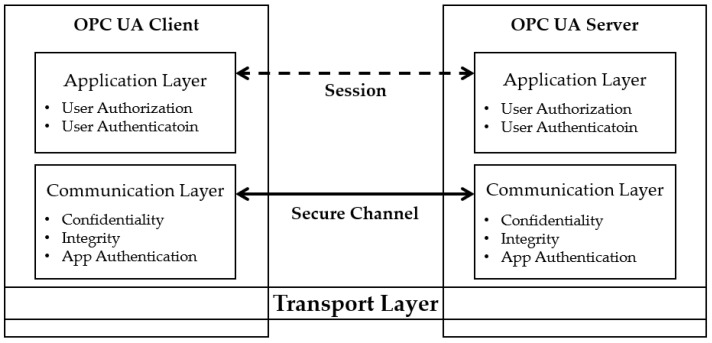
OPC UA security architecture.

Most security issues of OPC Classic are related to data access restrictions between DCOM and the transmission control protocol (TCP). The security of OPC Classic is set by the Windows DCOM-based user and authorization method for the user, which is difficult when OSs other than Windows are involved. The OPC UA is an interface between components at different levels of the industrial automation model, from high-level enterprise management to low-level field equipment control. Accordingly, the OPC Foundation provides a standard definition of the security of OPC UA, as presented in Figure 4 [2].

Transport layer: The lowest layer of the OPC UA security architecture uses a firewall to defend against external threats and rejects the connection itself. This layer also manages the IP addresses used by the server and client.

Communication layer: This layer provides confidentiality, integrity, and application authentication functions for security purposes. The OPC UA server and client negotiate security functions and create a secure channel. This layer transmits data after generating encryption to implement confidentiality, a signature to provide integrity, and a certificate of data received from the application layer.

Application layer: Communication, settings, and commands between client and server applications are provided in this layer. This layer is also responsible for user authentication and authorization for security purposes.

### 2.3. OPC UA Security Analysis

The security scope of the OPC UA is divided into reliable information and access control and is defined as the elements of the confidentiality, integrity, and availability (CIA) triad and the authentication, authorization, and accounting (AAA) framework. The OPC UA security architecture described in Section 2.2.3 is defined to achieve the security objective and is summarized in Table 4.

The security model defined in the OPC UA standard was implemented [20]. The authors defined the security requirements according to the OPC UA system construction environment, such as local networks, virtual networks, or Internet connections, and a distributed firewall-based security strategy was presented. The BSI reviewed the OPC UA security mechanisms and issued an evaluation report. The analysis report demonstrates that the OPC UA provides a high level of security, unlike other industrial protocols, and has no system errors based on the results of four security tests (certificate test, static code analysis, fuzzing, and dynamic code analysis). In addition, the *Practical Security Recommendations for Building OPC UA Applications* published by the OPC Foundation presents a guide on security modes, encryption algorithm selection, user authentication, certificate and private key storage, certificate usage, and certificate management and maintenance.

Nevertheless, safe OPC UA construction and operation are not performed in real industrial environments [6]. The study indicated that 92% of OPC UA servers had problems with security settings, such as missing access controls, disabled security mode, use of insecure encryption, and reuse of certificates. The authors suggested that the complexity of the OPC UA security settings was the cause and that the default values of the security configuration should be applied as a recommendation for OPC UA deployment. Other studies [14,21] that have assessed the security of OPC UA deployments have reported that the OPC UA standard protocol guarantees a high level of security, emphasizing the importance of correct security settings in OPC UA deployments.

Owing to the influence of the OPC UA security mechanism on performance [22,23], the use of message authentication and data encryption is not compulsory. This flexibility makes the OPC UA vulnerable to cyberattacks.

A security analysis of the OPC UA protocol was performed in [8,9]. In [8], most security vulnerabilities were due to products and libraries that did not meet the OPC UA standard specifications through fuzzing, and 17 security vulnerabilities in OPC UA products were identified. In [9], confidentiality and authentication properties were reviewed using ProVerif, an encryption protocol verification tool, and confidentiality and authentication requirements were satisfied when using the signing/encryption mode. Moreover, Erba et al. investigated 48 artifacts composed of products and libraries for the OPC UA and suggested that 38 of them had one or more security problems [10].

A study was conducted on an attack simulation for insecure OPC UA security configuration [11]. In addition, Varadarajan [12] focused on three major cyberattacks occurring in the industrial IoT [11]: packet sniffing, MiTM, and DoS. An attack scenario was constructed, and a penetration test was performed through a simulation [12]. Both studies verified the attack scenario through a cyberattack simulation that could occur in an insecure security configuration; however, it was only a penetration test for a single threat.

In addition, Hildebrandt et al. demonstrated that a command injection attack through a hidden channel could be performed on a packet transmitted from a server to a client (PLC) in an OPC UA protocol-based communication environment, a potential supply chain attack. Attack vectors have also been described [13]. For example, Polge et al. identified new threats that can occur using the OPC UA protocol based on IoT security threat modeling [14]. The attack identified from their proposed OPC UA threat model verified the possibility of two types of DoS attacks using MiTM and TCP, synchronization (SYN) flood attacks. Table 5 summarizes the corresponding studies and attack types and characteristics.

In this study, we propose a framework for analyzing vulnerabilities in the OPC UA protocol and modeling threats. We also present scenarios for vulnerabilities identified according to threat modeling and suggest countermeasures through the proof-of-concept process for each configured scenario.

### 2.4. Threat Modeling

The threat modeling phase is configured to perform three tasks. Based on the created data flow chart, threat modeling is applied to identify threats. When a threat is identified, a threat that can attack the analyzed target is derived. If common items among the derived threats are grouped and visualized, an attack tree, the basis of an attack scenario, can be created. Threat modeling typically includes the STRIDE [24,25,26], Process for Attack Simulation and Threat Analysis (PASTA) [27], Operationally Critical Threat Asset and Vulnerability Evaluation (OCTAVE) [28], Trike [29] and LINDDUN (linking, identifiability, nonrepudiation, detectability, disclosure of information, unawareness, and noncompliance) [30,31] methods.

1. STRIDE

Microsoft developed STRIDE, a security threat modeling method, in 1999. Threats of STRIDE include spoofing, tampering, repudiation, information disclosure, DoS, and elevation of privilege. The security attributes are displayed in Table 6.

2. PASTA

Next, PASTA is a threat modeling framework developed in 2012. The purpose of PASTA is to provide an attacker-centric view of the applications and infrastructure that defenders can use to develop asset-centric mitigation strategies. In addition, PASTA defines a seven-step process for identifying, enumerating, and scoring dynamic threats.

3. OCTAVE

The Software Engineering Institute at Carnegie Mellon University developed OCTAVE as a threat analysis and risk assessment methodology in 2003. The purpose of OCTAVE is to provide an operations-centric threat modeling method to evaluate organizational risks systematically. Further, OCTAVE identifies critical assets, threats, and vulnerabilities of assets necessary to conduct an organization’s business, which defines the process of developing a strategy to mitigate risks.

4. Trike

Trike is an integrated framework for security inspection from a risk management security perspective through the creation of threat models in a reliable and repeatable manner. It is a threat modeling technique that identifies users and assets in the data flow and usage flow and derives the risk to the asset by analyzing the frequency of user execution of the four elements of the asset: create, read, update, and delete.

5. LINDDUN

Next, LINDDUN addresses seven privacy-related threats and identifies the following: linkability, identifiability, non-repudiation, detectability, information disclosure, unawareness, and non-compliance. The LINDDUN threat model focuses on systematizing personal information threats by identifying external objects, processes, data storage, and data flows expressed in DFDs and representing each threat as a threat tree.

In the architecture standard described in OPC 10000-2, security threats to OPC UA systems are classified into 12 categories as 1. Denial of Service, 2. Eavesdropping, 3. Message spoofing, 4. Message alteration, 5. Message replay, 6. Malformed messages, 7. Server profiling, 8. Session hijacking, 9. Rogue Server, 10. Rogue Publisher, 11. Compromising user credentials, 12. Repudiation as follows. The security attributes affected by these threats can be countered, and compared to the scope of threat modeling listed in this paragraph. They are summarized in Table 7:

Each threat model has a different focus and analysis perspective. Therefore, selecting an appropriate threat modeling technique based on the target to be analyzed for vulnerabilities is important. For example, STRIDE is design-focused and focuses on software vulnerabilities. In contrast, PASTA focuses on requirement analysis and enterprise risk management assessment. OCTAVE is a threat modeling technique focusing on organizational risks, such as financial ones. Instead of evaluating and quantifying identified threats, Trike is used to classify threats in line with asset risk management. Like STRIDE, LINDDUN focuses on design, but it evaluates personal information. In this study, we focus on identifying vulnerabilities in the test bed using the OPC UA. Accordingly, STRIDE was employed as a threat modeling technique.

## 3. Vulnerability Discovery and Countermeasure Framework

### 3.1. Overview of the Vulnerability Discovery Methodology

*The Open-Source Security Testing Methodology Manual* (OSSTMM) [32], National Institute of Standards and Technology (NIST) SP800-115 [33], and Open Web Application Security Project (OWASP) [34] are the existing vulnerability discovery methodologies and include appropriate guidelines for penetration testing:

1. OSSTMM

The OSSTMM is one of the most widely used penetration testing standards developed by the Institute for Security and Open Methodologies. The OSSTMM provides detailed test plans, metrics to evaluate the current security level, and recommendations for creating a final report, ensuring that all tests are detailed and comprehensive. The OSSTMM proposes five main directions for operational security testing, as listed in Table 8.

2. NIST SP800-115

NIST SP800-115 provides an overview of the key elements of security testing. Technically, it provides a way to plan, conduct, analyze results, and develop remediation strategies for information security testing. This methodology includes the following:

  1. Inspection of documents, logs, system configuration, network sniffing, and file integrity;
  2. Evaluation of vulnerabilities through password cracking, social engineering, and penetration testing;
  3. Self-assessment of security through reconciliation, data processing, analysis, and evaluation;
  4. Post-assessment actions with recommendations for risk reduction, assessment reports, and vulnerability patching.

3. OWASP

The OWASP provides a methodology for testing applications, websites, and application programming interfaces (APIs). This document is useful for IT companies that intend to develop security software and includes the following:

  5. OWASP Top 10: A document describing the most well-known vulnerabilities in web and mobile applications, IoT, and APIs. Threats are described in terms of complexity and business impact.
  6. OWASP Testing Guide (TG): This document contains various techniques for testing web application security.
  7. OWASP Developer Guide: This guide provides recommendations for developing safe and reliable code.
  8. OWASP Code Review: This guide is distributed for use by web developers and product managers. It provides an effective method to test the security of existing code.

### 3.2. Proposed Vulnerability Discovery and Countermeasure Framework

**Figure 5:**
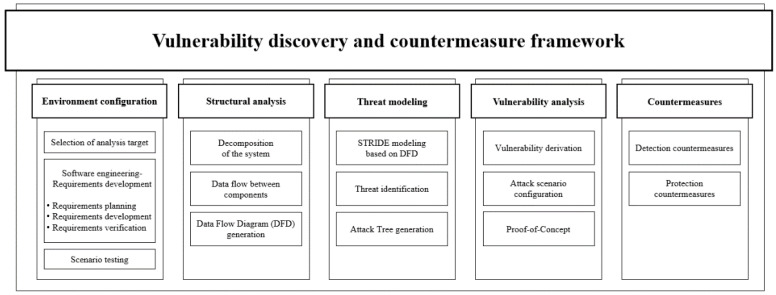
Proposed vulnerability discovery and countermeasure framework.

Discussed in Section 3.1, existing methods for discovering vulnerabilities perform analysis from the perspective of advanced persistent threat attacks as part of a penetration test. This study aims to discover vulnerabilities applicable to any analysis target and establish countermeasures. Therefore, the existing vulnerability discovery methodology cannot be applied; hence, a new framework is proposed. Figure 5 presents the vulnerability discovery and countermeasure framework proposed in this study, consisting of five steps. 

#### 3.2.1. Environment Configuration

The environment configuration consists of three steps. First, an analysis target is selected. The analysis target can be an aspect that can cause threat or risk, such as the corporate environment, smart factories, and smart devices. If the analysis target is abstract, a scenario including it can be constructed. In this case, requirement development and management techniques of software engineering are used. Usually, requirements in software engineering range from high-level abstract statements about system functions or compositions to detailed mathematical functional specifications. However, because the purpose of this framework is not to develop software, the requirements are interpreted as an analysis target.

Accordingly, scenarios are constructed through three processes. The first step in constructing a scenario is planning. It aims to plan the environment, including the object of the analysis. It also determines the type of system that the environment consists of. Then, it includes the functions that the system should include and what it does. The second step is development, which implements the plan. The third step is verifying that all conditions in the planning stage are met.

#### 3.2.2. Structural Analysis

The test scenario created through environmental configuration is structurally analyzed in the structural analysis stage. First, the system constituting the test scenario is disassembled. Through the decomposition of the system, major components, such as processes and external objects, can be represented, as presented in Table 9. Through this process, the data flow between entities can be identified. Finally, a data flow diagram (DFD) is created to gain visibility and identify threats.

#### 3.2.3. Threat Modeling

Threats are identified by applying STRIDE threat modeling based on the data flow diagram generated in the Structural Analysis step. It then analyzes the identified threats to create an attack tree on which the attack scenario is based. Thus, the threats identified by each entity were configured for use in an attack scenario. The attack tree produced by threat modeling determines which elements are needed for each attack from the main threats identified. Based on STRIDE threat modeling focused on software vulnerabilities, vulnerabilities are identified for each system component that attackers can exploit to compromise the entire system.

#### 3.2.4. Vulnerability Analysis

The vulnerability analysis stage uses a previously created attack tree. Possible vulnerabilities in the analysis target are deduced. Subsequently, an attack scenario that exploits the vulnerability in the analysis target is configured. The attack scenario should be configured according to the environment, including the analysis target determined in Step 1 of the framework. The validity of the vulnerability is verified through a proof-of-concept step that executes the attack scenario.

#### 3.2.5. Countermeasures

The last step is to establish a countermeasure against the attack performed in the previous step. First, the method of detecting and protecting using a function of the analyzed object is addressed. It is a countermeasure against the vulnerabilities verified in the previous step and may also be a countermeasure against the tools used in the attack scenario. The case study described in Section 4 suggests countermeasures according to each scenario.

## 4. Case Study

### 4.1. Environment Configuration

The target of the vulnerability analysis in this study is the OPC UA. Because the OPC UA is not an independent device or environment, it can be considered an abstract object. Therefore, a scenario that includes the OPC UA was configured. The first stage involves planning the environment including the OPC UA. The scenario of water and sewage facilities was implemented considering that the OPC UA is mainly used in an ICS environment as a control protocol.

**Figure 6:**
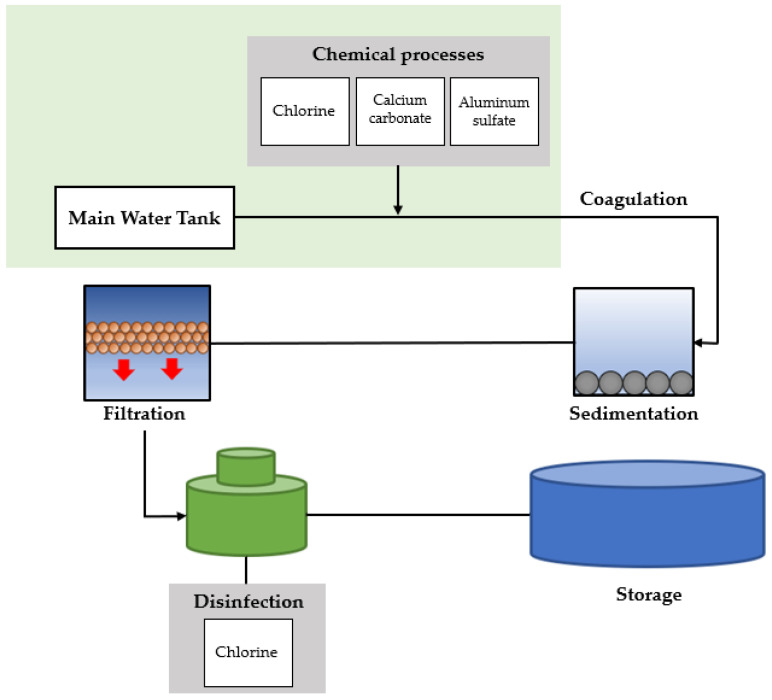
Water treatment process by the Pure Water Technology company.

The Pure Water Technology company is in charge of water purification in water and sewage. The water treatment process is performed as detailed in Figure 6. Water treatment refers to the process of purifying water. Generally, water is purified using chlorine disinfection and filtration methods. First, the water intake process is performed to obtain water from a water source. Chlorine, calcium carbonate, and aluminum sulfate are administered to the water to kill germs, eliminate odors, and settle solids in the water. The grains undergo coagulation and agglomeration to form large grains in the water in which the drug is administered. The agglomerated grains and water flow into the settling basin, where the grains settle. From the clarifier, the water flows through a filter of sand and gravel. Chlorine is again added as a disinfectant. Finally, the treated water is stored in reservoirs, called tanks or water tanks.

**Figure 7:**
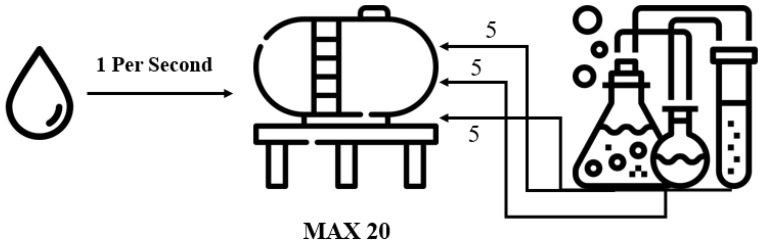
Water intake and chemical treatment phases.

Among the scenarios, the configuration for the water intake and chemical treatment steps was set, and the system environment was configured. Water intake and chemical processing are performed using automated on-site equipment. As illustrated in Figure 7, the main water tanks are filled with water at a rate of one tank per second, and after filling 20 water tanks, the chemical treatment stage is performed. In the chemical treatment stage, chlorine, calcium carbonate, and aluminum sulfate are added in amounts of five each, and if it is less than or more than five, water quality problems occur. The OPC UA protocol ensures interoperability because the equipment supplied for measuring the amount of water and the chemical processing equipment are different.

**Figure 8:**
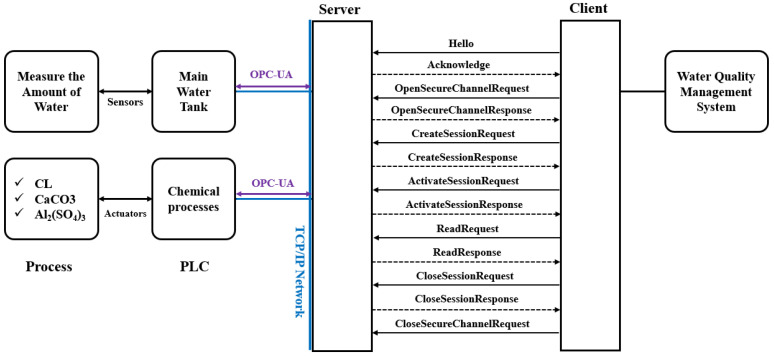
Connection structure between server–client and server–PLC in the scenario.

The server consists of a water tank and PLC in the chemical processing stage. As depicted in Figure 8, the water tank is connected to a sensor that measures the amount of water, and the chemical treatment PLC is connected to an actuator that injects chlorine, calcium carbonate, and aluminum sulfate. The client is connected to the water quality management system. The client receives information from the server about the amount of water in the tank and whether the chemical treatment has been implemented according to the set amount. The connection uses the OPC UA protocol, consisting of the GetEndPoint, OpenSecureChannel, CreateSession, and ActivateSession steps.

The OPC technology consists of a server and client. Therefore, to implement an environment that includes the OPC, it is necessary to implement a server and client. Table 10 lists the open-source implementations of the OPC UA.

Table 11 presents the requirements for the server–client implementation.

**Figure 9:**
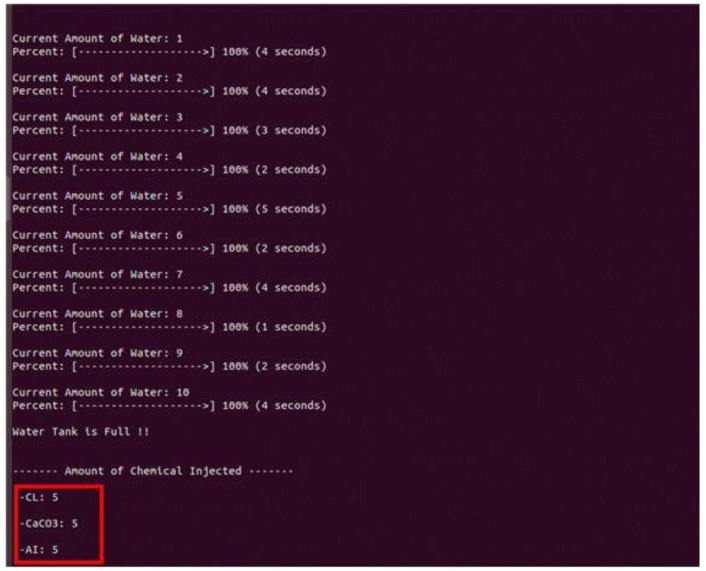
Scenario configuration using Python FreeOpcUa.

Table 12 lists the OPC UA open-source implementations that satisfy the requirements. The open-source implementations that satisfy all requirements are Nos. 2 and 5, and No. 5 was used to implement the scenario. A server–client pair was implemented in Linux using the Python FreeOpcUa open-source software. Figure 9 depicts the screen on which the scenario was executed using the open-source software.

### 4.2. Structural Analysis

**Figure 10:**
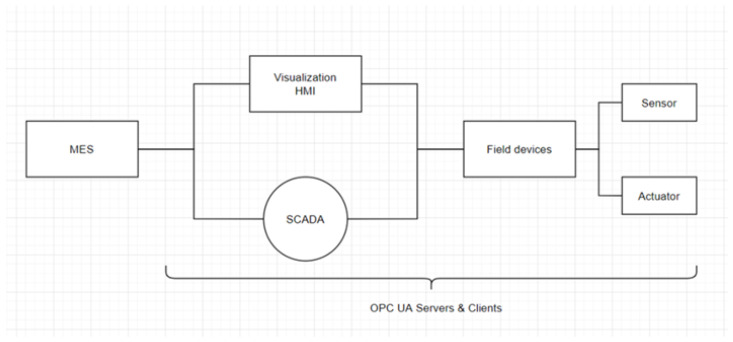
Configuration diagram for the scenario.

The configuration diagram for the scenario implemented in the environment configuration stage is presented in Figure 10.

The part where the server and client communicate with the OPC UA protocol is designated as the main object. The OPC UA operates as a server–client system. The server and client undergo authentication, secure channel opening, session creation, and session activation processes. The server and client exchange keys and tokens through requests and responses in each process. Accordingly, the entities comprise client and server certificates. The process consists of authentication, secure channel opening, session creation, and session activation. Table 13 summarizes the entities to be analyzed in the OPC UA.

**Figure 11:**
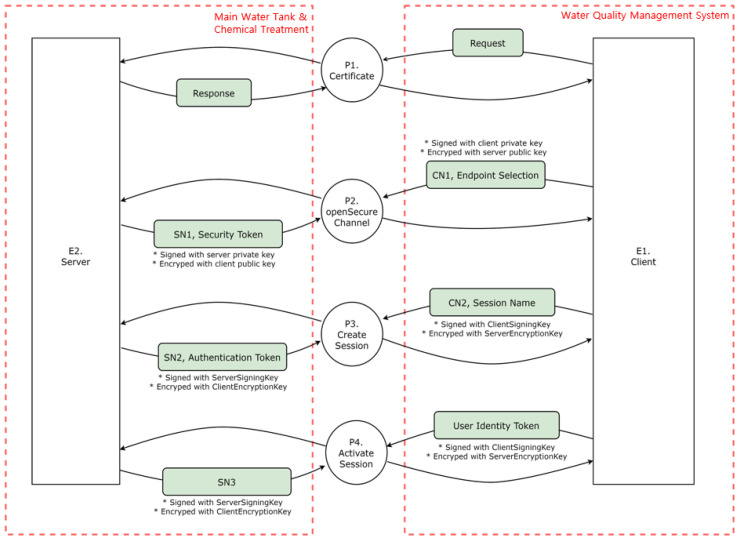
DFD of the OPC UA server–client system.

- P1 (Certificate): The server and client exchange requests and responses in the first process.
- P2 (Open secure channel): The client signs its private key through the OpenSecureChannel process, encrypts it with a public key, and sends it to the server. After receiving the transmission, the server signs its private key, encrypts it with a public key, and sends it to the client.
- P3 (Create session): After receiving the transmission, the server signs its private key, encrypts it with a public key, and sends it to the client. After creating a session, the client signs the client signing key, encrypts it with the server’s encryption key, and transmits it to the server. In addition, the server receives the message, signs the server’s signing key, encrypts the client’s encryption key, and delivers it to the client.
- P4 (Activate session): A user authentication token is sent to the server to activate a session, which the server receives when creating a session and sends it back to the client.
  A DFD is created based on identifying of the data flow between objects, as illustrated in Figure 11. The external objects are the server and client, and the main processes are authenticating, opening a secure channel, and creating and activating a session. The data flow of the OPC UA server–client system can be monitored by utilizing the Wireshark tool, which is discussed in Section 4.4.3, attack scenario proof-of-concept.

### 4.3. Threat Modeling

In this study, threat modeling was performed using the STRIDE technique. This threat model was proposed by Microsoft and has six goals of authentication, integrity, non-repudiation, confidentiality, availability, and authorization that provide information protection on the elements of spoofing, tampering, repudiation, information disclosure, DoS, and elevation of privilege. The threat modeling stage proceeds based on the DFD derived in the previous stage. Table 14 lists the threats according to the components of STRIDE based on the derived DFD.

This study uses the Microsoft Threat Modeling Tool v7.3.10801.1. The threats mapped to STRIDE can be automatically identified using the reporting function. Accordingly, 88 threats were derived from this analysis, and the main 30 threats are summarized in Table A1.

**Figure 12:**
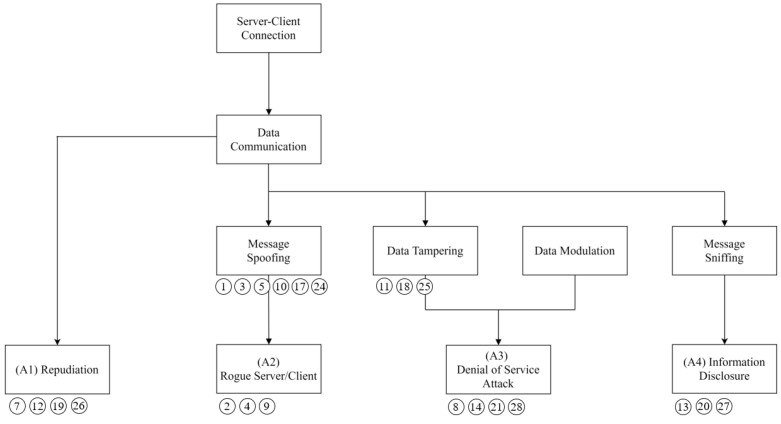
Derived attack tree.

Figure 12 demonstrates the attack tree generated based on the identified threats. Four attacks were derived based on the attack tree: repudiation, rogue server/client, DoS, and information disclosure.

### 4.4. Vulnerability Analysis

Step 4 is the vulnerability analysis. The vulnerability analysis proceeds with the vulnerability derivation, attack scenario configuration, and proof of concept.

#### 4.4.1. Vulnerability Derivation

The first step is to identify vulnerabilities. Table 15 presents descriptions of the attack types for the attack tree. An attack scenario was constructed based on attacks A1, A2, A3, and A4.

#### 4.4.2. Attack Scenario

The Pure Water Technology company is inspected by a maintenance company once a month. An employee of the maintenance company introduces a script that captures packets at regular intervals in the system of the Pure Water Technology company through a USB connection to the internal system for sabotage. In the internal system, the script in the USB is automatically executed to capture packets at regular intervals. After a month, the maintenance staff analyzes the packets by placing the packet capture file on a USB. The network configuration diagram, IP address, and system port are determined through the analysis, and an attack is executed using them. The attack scenarios consist of message manipulation by impersonating clients and DoS attacks through flooding attacks. We address the first scenario, manipulating messages through impersonated clients. The files comprising the server and client are listed in Table 16.

The attack scenario process of Scenario 1 is as follows:

1. The server runs opcuaServer.py to open port 4840 and waits for a client connection.
2. A normal client communicates with the server through socket communication and exchanges keys using the Diffie–Hellman algorithm.
3. The rogue client detects the secret key between the server and the normal client.
4. The rogue client exchanges the key with the server through the detected secret key and then engages in the authentication process through the forged certificate.
5. After the connection process is complete, the rogue client manipulates the sensor and actuator values of the server into abnormal values to perform an attack.

Scenario 2 is a DoS attack through a flooding attack, which is an attack scenario that causes a DoS attack by attempting multiple connections while the server remains in a listening state. In addition, hping3 was employed as the attack tool. The parameters used in hping3 and their descriptions are summarized in Table 17. The IP addresses and statuses of attackers and victims are presented in Table 18.

#### 4.4.3. Proof of Concept

**Figure 13:**
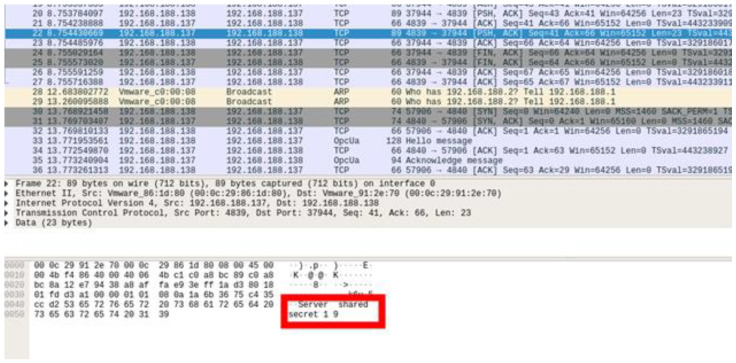
Rogue client eavesdrops on the key exchange process (capturing the “Server shared secret 19” packet).

First, a proof of concept was conducted for message manipulation through the spoofed client in Scenario 1. Before executing a rogue client, an attacker uses eavesdropping. The attacker employs Wireshark to capture packets exchanged between the server and client. The key exchange process in the first step can be viewed as plain text, and, as indicated in Figure 13, the rogue client can determine the symmetric key. The second step in implementing a rogue client is certificate manipulation. The certificate used by the OPC UA is X.509, and anyone can create it using OpenSSL. Both “my_cert.der” and “client_private_key.pem” were created using OpenSSL for authentication. If the authentication is successful, then the server and client start communication.

**Figure 14:**
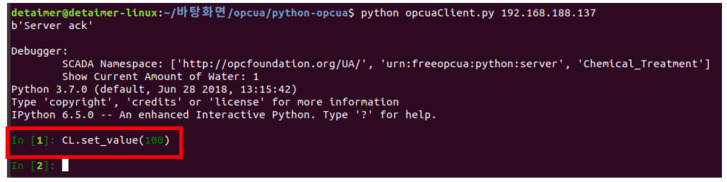
Rogue client manipulates values (normal value: 10; manipulated value: 100).

**Figure 15:**
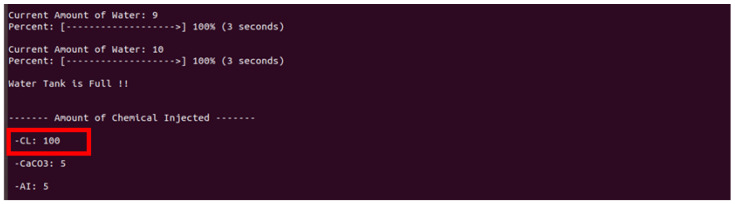
Screen manipulated by a rogue client.

If the connection is successful, the rogue client can observe a screen similar to that in Figure 14 and directly input commands to manipulate the values (Figure 15).

Figure 15 shows the changed result when the rogue client in Figure 14 executes the command to inject 100 chlorine, which is 10 times the normal value.

**Figure 16:**
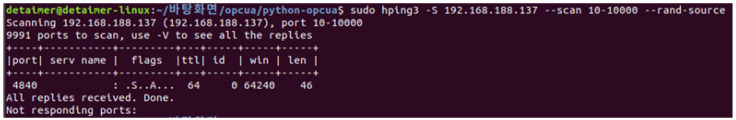
Attacker check open ports.

Second, a proof of concept for the DoS attack through the flooding attack, Scenario 2, was conducted. The process in Scenario 2 begins with port scanning using hping as shown in Figure 16:

1. Port scan step: Scan open ports using the scan parameter.

2. The SYN flooding attack using hping3: execute the SYN flooding attack using the following command.

ubuntu@ubuntu-linux:~$ sudo hping3 –S 192.168.188.142 –p 4840

**Figure 17:**
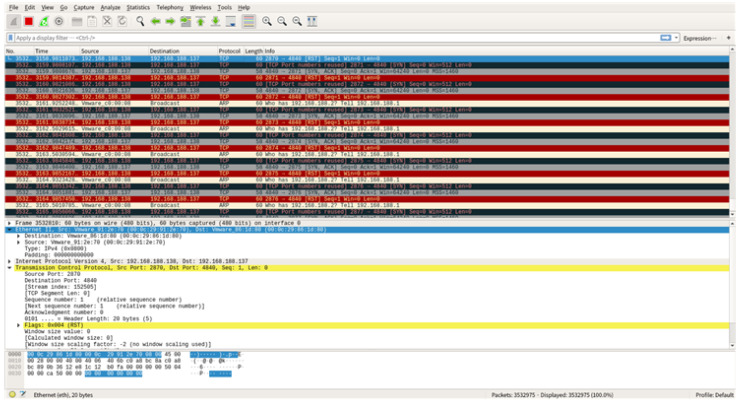
Wireshark screen of the victim under an SYN flooding attack.

Through the attack, the server continuously receives packets with the SYN flag, the set waiting queue becomes full, and availability is lost. Figure 17 reveals that a packet with the SYN flag is specified to the source IP address (192.168.188.138) through Wireshark from the point of the victim (192.168.188.137).

### 4.5. Countermeasures

Finally, employing the security function or attack detection method in the analysis target was suggested as a countermeasure. The countermeasures for attack Scenario 1 are as follows:

1. Using of encryption algorithms

OPC UA uses encryption to ensure confidentiality for security. Symmetric encryption protects all messages transmitted between OPC UA applications, and asymmetric encryption is conducted through key exchange. The algorithm presented in Table 19 is recommended for the OPC UA security policy [42].

2. Certificate management and distribution

Two functions exist in the OPC UA for certificate management and distribution. First, all entities communicating in the OPC UA network establish a trusted certificate list (certificate trust list). Second, application authentication is executed using the CertificateManager function. For further details, refer to *6.1.3 Determining if a Certificate is Trusted* from OPC 10000-4 [43]. An application in the OPC UA must determine whether to trust another application instance certificate by verifying whether it can be trusted. The evaluation criteria include a list of trusted applications and a list of trusted certification authorities. If the application cannot be trusted directly (the certificate is not on the list of trusted applications), then the certificate chain must be rebuilt with a trusted certificate authority. Establishing a chain of trust requires access to all certificates in the chain, which are stored locally or with the certificate authority. Table A2 specifies the steps taken to validate the certificate for compliance.

The following are countermeasures for attack Scenario 2.

1. OpenSecureChannel request control

Due to server signing and encryption processing, responses to OpenSecureChannel require significant server resources. Therefore, most DoS attacks occur at the OpenSecureChannel service level. Therefore, two methods have been proposed on the server side. First, the server may intentionally delay the OpenSecureChannel request processing if it receives malicious OpenSecureChannel requests exceeding the specified minimum value. In addition, it sets an alarm indicating that a malicious request has occurred and sends it to the administrator. Second, when an OpenSecureChannel request attempts to exceed the number of simultaneous access channels specified by the server, the server sends an error response without performing signature and encryption processing. An authorized OPC UA server shall specify the maximum number of simultaneous channels specified in OPC 10000-7 [44].

2. Authenticated client

An unauthenticated client performs a flooding attack to cause DoS attacks. Therefore, it is possible to apply the recommended guideline for the session activation service of OPC 10000-4. The client uses session activation to specify the ID of the user associated with the session. The client must request the main service before generating service requests other than session creation and termination. When a client calls this service, it must prove that it is the same application that called the session creation service. The client performs the verification process by signing with a private key associated with the client certificate specified in the session creation request. The nonce received from the server is added to the server certificate, and the byte order is calculated to generate a signature. Because the server creates a new nonce every time the session activation service is called and sends it to the client, the old nonce cannot be reused. When the session activation service is called and the secure channel is not related to the session creation request, the server rejects the session activation service request. Subsequent calls for session activation can connect to other secure channels. In this case, the server must ensure that the certificate used by the client to create the new secure channel is the same as that used to create the current secure channel. In addition, the server must ensure that the client’s user ID is the same as that associated with the current session.

The session activation service associates a user ID with a session. When a client provides a user ID, it must prove that it is authorized to use it. The mechanism used to provide this evidence depends on the type of user identity. In the case of UserNameIdentity, it is the mechanism that contains the token; in the case of X509Identity, the token is a signature generated by the private key associated with the certificate. The data to be signed are written by adding the nonce received from the server to the serverCertificate. If the token requires encryption, it must be encrypted using the public key of the certificate. Servers must take appropriate measures to protect against attacks on user ID tokens. An attack occurs when repeated connection attempts are made using a malformed user ID token. A workaround is to lock the OPC UA client for a certain period if the user ID token validation fails multiple times. The OPC UA clients detect unsecured connections via IP addresses or secure connections using ApplicationInstanceUri. Another measure is to delay the service response if the user ID validation fails.

### 4.6. Compare the Conventional and Proposed Method

Comparing the conventional method described in Chapter 3 and the method proposed in this study, it can be summarized as shown in Table 20. Conventional methods identify vulnerabilities in general IT environments or web applications. In the method proposed in this study, vulnerability discovery and countermeasure steps are performed in terms of OT systems, including industrial IoT.

## 5. Conclusions

The number of threats discovered yearly is increasing, and it is critical to detect them in advance. Therefore, configuring the environment in which one wants to discover threats and vulnerabilities and identify countermeasures is necessary. In this paper, a framework for vulnerability discovery and response was presented. Existing vulnerability discovery frameworks have limited analysis targets. The proposed framework can be applied to any analysis target and comprises five steps. The OPC UA was selected as the analysis target for applying the framework in this study, and a case study was conducted. First, a scenario that included the analysis target was constructed. Then, a structural analysis was conducted. We created a DFD to explain the data flow through the structural analysis.

Next, in the threat modeling stage, we identified 30 major threats that could occur based on the DFD. The attack tree was created by grouping the most common of the 30 threats. Several threat modeling techniques can be used in the threat modeling stage, and the STRIDE technique was applied in this study. In the vulnerability analysis phase, possible attack scenarios were constructed using an attack tree. A rogue client attack using certificates and a DoS attack using flooding were constructed and validated through an actual proof of concept. The final step is to develop countermeasures, which include leveraging the capabilities of the target being analyzed to detect and protect it. In the future, we aim to validate vulnerabilities using other analysis targets or validate targets in other areas.

## Figures and Tables

**Table 1:** Difference between IT and an ICS.

	IT	ICS
Configuration environment	- Standardized equipment (PC, server) - Short equipment replacement cycle - Easy to patch and repair - Use a universal operating system - Network speed and performance	- Specialized equipment according to the process - Few equipment replacement cycles - Difficult to patch and repair due to equipment availability - General-purpose OS customized (e.g., embedded and kernel) or self-developed OS operation - Real-time network communication is important
Critical security objectives	- Block the leakage of important data and service interruption	- Block the possibility of production and process disruption - Prevention of personal accidents in case of accidents
Effect on security threats	- Damage caused by leaking important data - Legal issues and damage to company trust	- Direct damage caused by production and human casualties - Damage to product reliability

**Table 2:** Types and characteristics of major control protocols.

Industrial Control Protocol	Protocol Characteristics
PROFINET	Abbreviation for process field net, an open industry standard Ethernet-based protocol built and maintained by PROFINET International (PI)
Siemens S7	Siemens PLC control protocol
Modbus	PLC open standard serial communication protocol developed by Modicon
OPC-DA	Protocol for real-time data communication between client and server
OPC UA	Machine-to-machine communication protocol for industrial automation

**Table 3:** Analysis of the security elements of key control protocols.

Industrial Control Protocol	Encryption	Authentication	Security Features at Application Level
Modbus-TCP	-	-	Modbus (No security)
DNP3	Secure DNP	Secure DNP	Secure DNP
PROFINET	-	-	-
OPC	OPC UA	OPC UA	OPC UA
S7Comm	-	-	-

**Table 4:** OPC UA security requirements.

	Security Purposes	Implementation
Reliable information (CIA triad)	Confidentiality	Encryption at the transport layer
	Integrity	Signing at the transport layer
	Availability	Message size limit
Access control (AAA framework)	Authentication	Use of X.509 certificates and user account-based authentication at the application layer
	Authorization	User role-based access control
	Accounting	Generate audit events for security-related actions

**Table 5:** Summary of papers related to OPC UA penetration testing.

	Author	Year	Attack Type	Description
[8]	Pavel Cheremushkin et al.	2018	DoS, Remote code execution	OPC UA penetration testing using fuzzing techniques
[9]	Puys, Maxime et al.	2016	Privilege escalation	Validation of OPC UA confidentiality and security of authentication attributes using a cryptographic protocol verification tool
[10]	Erba, Alessandro et al.	2021	MiTM	Security evaluation and vulnerability attack verification for commercial OPC UA products
[11]	Neu, Charles Varlei et al.	2019	DoS	Implementation and evaluation of DoS attack scenarios for OPC UA clients not using the secure mode
[12]	Varadarajan, Vaishnavi	2022	Packet sniffing, MiTM, DoS	Implementing attack simulations for the three most common types of attacks in the IoT
[13]	Hildebrandt, Mario et al.	2020	Supply chain attack	Supply chain attack verification through the OPC UA server–client hidden channel
[14]	Polge, Julien et al.	2019	MiTM, message flooding	OPC UA threat modeling and attack scenario implementation based on IoT threat modeling

**Table 6:** Attributes and description of the threat modeling method STRIDE.

STRIDE	Security Attributes	Description
Spoofing	Authentication	Acquiring privileges using an illegal account
Tampering	Integrity	Illegal changing of data
Repudiation	Non-repudiation	Disclaiming failure to perform certain services or disclaiming liability
Information disclosure	Confidentiality	Giving information to someone who does not have access
DoS	Availability	Preventing a service or application from performing normally
Elevation of privilege	Authorization	Authorizing someone to perform an unauthorized service

**Table 7:** Comparison of the scope of OPC UA and threat modeling in response to security objectives.

	Object	Confidentiality	Integrity	Availability	Authentication	Authorization	Auditability	Non-Repudiation
Threat								
OPC UA		2, 7, 8, 9, 10, 11	3, 4, 6, 7, 8, 9	1, 6, 7, 8, 9,10	2, 4, 5, 7, 8, 9, 10, 11	2, 3, 4, 5, 7, 8, 9, 10, 11	4, 7, 8, 9, 10	4, 7, 8, 12
STRIDE		I	T	D	E	S	-	R
Trike		Elevation of Privilege	Elevation of Privilege	Denial of Service		Elevation of Privilege		
OCTAVE		-	-	-	-	-	-	-
LINDDUN		Disclosure of information (D), Detectability (D)	-	-	Identifiability(I), Linkability(L)	Identifiability(I)	Non-compliance (N)	Non-repudiation (N)
PASTA		-	-	-	-	-	-	-

**Table 8:** Types and characteristics of major control protocols.

Main Direction	Description
Human security	Security aspects that deal with direct physical or psychological interactions between people
Physical security	A security aspect that covers all material elements of security, whether physically or electromechanically actuated
Wireless communications	Security of all wireless communications and devices from Wi-Fi to infrared sensors
Telecommunications	Tests all communications over the network, whether the communications network is digital or analog
Data networks	Data network security testing involves electronic systems and data networks used to communicate or interact over cable and wired network lines

**Table 9:** Components of a DFD.

Component	Symbol	Description
External entity	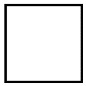	External objects create data inputs and check outputs
Data store	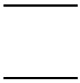	Data stores store data temporarily or permanently
Process	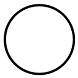	Processes are responsible for taking data input and generating output
Data flow	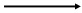	Data flow refers to the movement of data between objects
Trust boundary	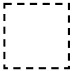	Trust boundaries represent changes in privilege levels

**Table 10:** OPC UA open-source implementation list.

No.	Name	Language	Client/Server
1	Open62541 [35]	C	Client and server
2	UA.NET Standard [36]	C#	Client and server
3	node-opcua [37]	JavaScript	Client and server
4	FreeOpcUa [38]	C++	Client and server
5	Python FreeOpcUa [39]	Python	Client and server
6	OpenScada UA Interface [40]	C++	Server
7	ASNeG [41]	C++	Client and server

**Table 11:** Server–client requirement features.

No.	Server	Client
①	Connection with the server (Channel and session)	Create channels and sessions
②	View and read property values	Get the path and node
③	Add/remove nodes	Events
④	Call method	Methods
⑤	Username/password	Encryption
⑥	Certificate login	Certificate handling
⑦	Communication encryption	Change data

**Table 12:** Checklist for open-source implementations that meet the requirements.

Open-Source List	Server							Client						
	①	②	③	④	⑤	⑥	⑦	①	②	③	④	⑤	⑥	⑦
Open62541	✓	✓	✓	✓	-	-	✓	✓	✓	✓	✓	✓	-	✓
UA.NET Standard	✓	✓	✓	✓	✓	✓	✓	✓	✓	✓	✓	✓	✓	✓
node-opcua	✓	✓	✓	✓	✓	✓	✓	✓	✓	✓	✓	✓	✓	✓
FreeOpcUa	✓	✓	✓	✓	-	-	-	✓	✓	✓	✓	-	-	✓
Python FreeOpcUa	✓	✓	✓	✓	✓	✓	✓	✓	✓	✓	✓	✓	✓	✓
OpenScada UA Interface	✓	✓	✓	✓	✓	✓	✓	-	-	-	-	-	-	-
ASNeG	✓	✓	✓	✓	-	-	-	✓	✓	✓	✓	-	-	✓

**Table 13:** Entities to be analyzed in the OPC UA.

Element	Name	Sign
External object	Client	E1
	Server	E2
Process	Certificate	P1
	Open Secure Channel	P2
	Create Session	P3
	Activate Session	P4

**Table 14:** Threats available in DFD components.

Threat	S	T	R	I	D	E
External entity	✓					✓
Data flow	✓	✓	✓	✓	✓	✓

**Table 15:** Attack types and descriptions based on the attack tree.

Attack Types	Attack Descriptions
A1	Creates trust issues through denial of data by the sender and receiver
A2	Manipulates clients by stealing credentials
A3	An untrusted client/server exists on the same network and continuously sends messages to flood the network and OPC UA server to perform a DoS attack
A4	Steals server/client information

**Table 16:** Attack types and attack descriptions based on the attack tree.

	Server	Client
Python file	opcuaServer.py	opcuaClient.py
Certification	certificate-example.der	my_cert.der
	private-key-example.pem	client_private_key.pem
Symmetric key storage file	serverkey.txt	serverkey.txt
	client.txt	client.txt

**Table 17:** Main parameters and their descriptions for hping3.

Parameters	Descriptions
-S	SYN flag setting
<IP_ADDRESS>	Destination IP
--scan	SCAN mode
-p	Set destination port number
--rand-source	Send source IP randomly
--flood	Sending many packets in a short time

**Table 18:** IP address and status of the attacker and victim.

	Attacker	Victim
IP Address	192.168.188.143	192.168.188.142
Condition	Attack using hping3	Listening status

**Table 19:** OPC UA security policy algorithm.

Algorithm Name	Description
PolicyUri	URI assigned to the security policy
SymmetricSignatureAlgorithm	Symmetric signature algorithm
SymmetricEncryptionAlgorithm	Symmetric encryption algorithm
AsymmetricSignatureAlgorithm	Asymmetric signature algorithm
AsymmetricEncryptionAlgorithm	Asymmetric encryption algorithm
MinAsymmetricKeyLength	Minimum length of the asymmetric key
MaxAsymmetricKeyLength	Maximum length of the asymmetric key
KeyDerivationAlgorithm	Key derivation algorithm
DerivedSignatureKeyLength	Bit length of the derived key to authenticate a message
CertificateSignatureAlgorithm	Asymmetric signing algorithm to sign certificates
SecureChannelNonceLength	Length (in bytes) of nonces exchanged when creating a secure channel

**Table 20:** Compare the conventional and proposed method.

	Conventional Method		Proposed Framework
	OSSTMM	OWASP-TG	
Testing Steps	6 phases	5 phases	5 phases
Countermeasure	X	O	O
Features	Applicable to IT systems	Applicable to Web	Applicable to operational technology (OT) system

## Data Availability

Not applicable.

## Appendix A

**Table A1:** Main threats by OPC UA scenarios.

Element	Element Name	STRIDE	Threat Analysis	Threat Number
External Entity	Client	S	Client may be spoofed by an attacker and this may lead to unauthorized access to Process. Consider using a standard authentication mechanism to identify the external entity.	T1
		E	Clients may be able to execute code for a process remotely.	T2
	Server	S	Server may be spoofed by an attacker and this may lead to unauthorized access to Process. Consider using a standard authentication mechanism to identify the external entity.	T3
		E	Server may be able to remotely execute code for Process.	T4
	Certificate	S	Certificate may be spoofed by an attacker and this may lead to information disclosure by external entity. Consider using a standard authentication mechanism to identify the destination process.	T5
		T	Data flowing across a response may be tampered with by an attacker. This may lead to a DoS or elevation-of -privilege attack against certificate or information disclosure by the certificate. Failure to verify that input is as expected is a root cause of numerous exploitable issues. Consider all paths and the way they handle data. Verify all input using an approved list input validation approach.	T6
		R	The certificate claims it did not receive data from a source outside the trust boundary. Consider logging or auditing to record the source, time, and summary of the received data.	T7
		D	The certificate crashes, halts, stops, or runs slowly; in all cases violating an availability metric.	T8
		E	The certificate may impersonate the context of an external entity to gain additional privilege.	T9
	Open Security Channel	S	OpenSecure Channel may be spoofed by an attacker, leading to information disclosure by an external entity. Consider using a standard authentication mechanism to identify the destination process.	T10
		T	Data flowing across the CN1, endpoint selection, SN1, and Security token may be tampered with by an attacker, leading to a DoS or elevation-of-privilege attack against the OpenSecure Channel or an information disclosure by OpenSecure Channel. Failure to verify that input is as expected is a root cause of numerous exploitable issues. Consider all paths and the way they handle data. Verify all input using an approved list input validation approach.	T11
		R	The OpenSecure Channel claims that it did not receive data from a source outside the trust boundary. Consider logging or auditing to record the data source, time, and summary.	T12
		I	Data flowing across the CN1, endpoint selection, SN1, security token may be sniffed by an attacker. Depending on what type of data an attacker can read, data may be used to attack other system parts or be disclosed, leading to compliance violations. Consider encrypting the data flow.	T13
		D	The OpenSecure Channel crashes, halts, stops, or runs slowly; in all cases violating an availability metric.	T14
		E	The OpenSecure Channel may impersonate the context of an external entity to gain additional privilege.	T15
		E	An attacker may pass data into the OpenSecure Channel to change the program execution flow to the attacker’s choice.	T16
	Create Session	S	Create session may be spoofed by an attacker, leading to information disclosure by an external entity. Consider using a standard authentication mechanism to identify the destination process.	T17
		T	Data flowing across the CN2, session name, SN2, and authentication token may be tampered with by an attacker, to a DoS or elevation-of-privilege attack against Create Session or an information disclosure by Create Session. Failure to verify that input is as expected is a root cause of numerous exploitable issues. Consider all paths and the way they handle data. Verify all input using an approved list input validation approach.	T18
		R	Create Session claims it did not receive data from a source outside the trust boundary. Consider logging or auditing to record the data source, time, and summary.	T19
		I	Data flowing across the CN2, session name, and SN2, authentication token may be sniffed by an attacker. Depending on what type of data an attacker can read, it may be used to attack other parts or be disclosed, leading to compliance violations. Consider encrypting the data flow.	T20
		D	Create Session crashes, halts, stops, or runs slowly; in all cases violating an availability metric.	T21
		E	An attacker may pass data into Create Session to change the program execution flow to the attacker’s choice.	T22
		E	An attacker may pass data into Create Session to change the program execution flow to the attacker’s choice.	T23
	Activate Session	S	Activate Session may be spoofed by an attacker, and this may lead to information disclosure by an external entity. Consider using a standard authentication mechanism to identify the destination process.	T24
		T	Data flowing across the SN3, and user identity token may be tampered with by an attacker, to a DoS or elevation-of-privilege attack against Activate Session or an information disclosure by Activate Session. Failure to verify that input is as expected is a root cause of numerous exploitable issues. Consider all paths and the way they handle data. Verify all input using an approved list input validation approach.	T25
		R	Activate Session claims it did not receive data from a source outside the trust boundary. Consider logging or auditing to record the data source, time, and summary.	T26
		I	Data flowing across SN3, User Identity Token may be sniffed by an attacker. Depending on what type of data an attacker can read, it may be used to attack other system parts or be a disclosure of information leading to compliance violations. Consider encrypting the data flow.	T27
		D	Activate Session crashes, halts, stops, or runs slowly; in all cases violating an availability metric.	T28
		E	Activate Session may impersonate the context of an external entity to gain additional privilege.	T29
		E	An attacker may pass data into Activate Session to change the program execution flow to the attacker’s choice.	T30

**Table A2:** Certificate management and deployment steps.

Stages	Error/Audit Event	Descriptions
Certificate structure	- Bad_CertificateInvalid - Bad_SecurityChecksFailed - AuditCertificateInvalidEventType	- Check the certificate structure - No error suppression
Build certificate chain	- Bad_CertificateChainIncomplete - Bad_SecurityChecksFailed - AuditCertificateInvalidEventType	- Create a certificate trust chain - Errors may occur during chain creation
Signature	- Bad_CertificateInvalid - Bad_SecurityChecksFailed - AuditCertificateInvalidEventType	Reject the certificate if the signature is invalid and the issuing authority is unknown
Security policy check	- Bad_CertificatePolicyCheckFailed - Bad_SecurityChecksFailed - AuditCertificateInvalidEventType	Certificate signing complies with the certificate signing algorithm and asymmetric key length algorithm for the user security policy defined in OPC 10000-7
Trust list check	- Bad_CertificateUntrusted - Bad_SecurityChecksFailed - AuditCertificateUntrustedEventType	If the application instance certificate is not trusted and the certificate authority in the chain is not trusted, the certificate validation result will fail
Validity period	- Bad_CertificateTimeInvalid - Bad_CertificateIssuerTimeInvalid - AuditCertificateExpiredEventType	Must be within the validity period of the certificate
Host name	- Bad_CertificateHostNameInvalid - AuditCertificateDataMismatchEventType	- The hostname in the URL to connect to the server is the same as that of the hostname specified in the certificate - Certificate authority certificates skip this step
URI (Uniform Resource Identifier)	- Bad_CertificateUriInvalid - AuditCertificateDataMismatchEventType	- Application and software certificates contain an application or product URI that must match the URI specified in the application description provided with the certificate - Certificate authority certificates skip this step - GatewayServerUri is used to validate the application certificate when connecting to the gateway server
Certificate usage	- Bad_CertificateUseNotAllowed - Bad_CertificateIssuerUseNotAllowed - AuditCertificateMismatchEventType	Each certificate must match its intended use (OPC 10000-6)
Find revocation list	- Bad_CertificateRevocationUnknown - Bad_CertificateIssuerRevocationUnknown - AuditCertificateRevokedEventType	Each certificate authority certificate has a revocation list, but this step fails if that list is not available
Revocation check	- Bad_CertificateRevoked - Bad_CertificateIssuerRevoked - AuditCertificateRevokedEventType	The certificate has been revoked and cannot be used
